# Resistance to Anti-angiogenic Therapies: A Mechanism Depending on the Time of Exposure to the Drugs

**DOI:** 10.3389/fcell.2020.00584

**Published:** 2020-07-07

**Authors:** Christopher Montemagno, Gilles Pagès

**Affiliations:** ^1^Département de Biologie Médicale, Centre Scientifique de Monaco, Monaco, Monaco; ^2^CNRS UMR 7284, Institute for Research on Cancer and Aging of Nice, Université Côte d’Azur, Nice, France; ^3^INSERM U1081, Centre Antoine Lacassagne, Nice, France

**Keywords:** VEGFA, anti-angiogenic treatments, resistance, tumor microenvironment, combined therapies

## Abstract

Angiogenesis, the formation of new blood vessels from preexisting one, represents a critical process for oxygen and nutrient supply to proliferating cells, therefore promoting tumor growth and metastasis. The Vascular Endothelial Growth Factor (VEGF) pathway is one of the key mediators of angiogenesis in cancer. Therefore, several therapies including monoclonal antibodies or tyrosine kinase inhibitors target this axis. Although preclinical studies demonstrated strong antitumor activity, clinical studies were disappointing. Antiangiogenic drugs, used to treat metastatic patients suffering of different types of cancers, prolonged survival to different extents but are not curative. In this review, we focused on different mechanisms involved in resistance to antiangiogenic therapies from early stage resistance involving mainly tumor cells to late stages related to the adaptation of the microenvironment.

## Introduction

**FIGURE 1:**
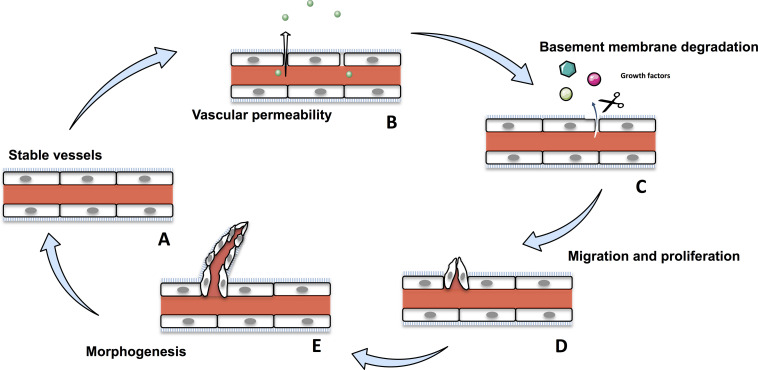
The physiological angiogenic cascade. Stable vessels **(A)** undergo a vascular permeability allowing extravasation of plasma proteins **(B)**. MMPs degrate the extracellular matrix liberating growth factors **(C)**. ECs proliferate and migrate **(D)** and undergo morphogenesis and assemble as lumen-bearing cords **(E)**.

Angiogenesis is the formation of new blood vessels from pre-existing ones (Hanahan and Folkman, 1996). It is a crucial physiological process that occurs throughout the life time, from the embryo to establish an adequate vasculature for growing and developing organs, to adults during wound healing or ovarian cycle (Folkman and Shing, 1992; Wilting and Christ, 1996; Hazzard and Stouffer, 2000; Tonnesen et al., 2000). Angiogenesis is tightly regulated and disruption of any part of this process induces various disorders, such as psoriasis, diabetic retinopathy, and cancer (Nishida et al., 2006; Walsh, 2007; Crawford et al., 2009; Heidenreich et al., 2009). Angiogenesis involves migration, proliferation and differentiation of endothelial cells (ECs). During the angiogenic cascade, stable vessels undergovascular permeability and a basement membrane degradation by the matrix-metalloproteases (MMPs) liberating extracellular matrix-sequestred growth factors. In response to these growth factors, ECs proliferate and migrate to assemble as lumen-bearing cords with branching structure (Figure 1; Bryan and D’Amore, 2007). Angiogenesis is a tightly balanced mechanism regulated by both pro-angiogenic and anti-angiogenic factors. In tumors, this balance shift toward pro-angiogenic factors sustaining angiogenesis.

One of the first relationships between angiogenesis and cancer was introduced 55 years ago when Ehrmann and Knoth (1968); Greenblatt and Shubi (1968), P highlighted for the first time, that tumors secrete substances targeting ECs that stimulate angiogenesis. Three years later, Judah Folkman observed that the growth of solid tumors relies on this process (Folkman, 1971, 1972). The newly formed vascular network supplies tumor with oxygen, nutrients and growth factors. Based on these observations, Folkman proposed that inhibiting angiogenesis through ECs inhibition should constitute a promising anti-cancer treatment, by preventing nutrients supply and oxygen to tumors. This original concept stipulated that ECs are normal cells incapable of genetic plasticity as compared to tumor cells. Therefore, destruction of the blood vessel should have lead to tumor cell asphyxia and thereafter complete tumor regression. This concept was confirmed by the discovery of several angiogenic factors, such as transforming growth factor-α and β (TGF-α and TGF-β), angiopoietin, epidermal growth factor (EGF), platelet-derived growth factor (PDGF) and vascular endothelial growth factor (VEGF)A (Schreiber et al., 1986; Ferrara and Henzel, 1989; Levéen et al., 1994; van Cruijsen et al., 2005; van Meeteren et al., 2011; Fagiani and Christofori, 2013).

In 1989, the discovery of VEGFA, one of the most important angiogenic factors, by independent teams was a real breakthrough in understanding the mechanisms of angiogenesis (Keck et al., 1989; Leung et al., 1989; Guyot and Pagès, 2015). Four years later, the first monoclonal neutralizing antibody directed against VEGFA was described by the team of N. Ferrara, the winner of the Lasker Award few years laters for the use of these antibodies in eye pathologies especially wet age-related macular degeneration (Kim et al., 1993). This antibody inhibited the growth of experimental models of rhabdomyosarcoma, glioblastoma and colorectal and prostate cancers (Kim et al., 1993; Asano et al., 1995; Warren et al., 1995; Borgström et al., 1998). These promising anti-tumoral effects led to the development of bevacizumab (Avastin^®^), a humanized anti-VEGFA monoclonal antibody (Presta et al., 1997). Bevacizumab by specifically inhibiting the binding of VEGFA to its receptor VEGFR2 present on ECs, blocks signaling pathways involved in ECs proliferation and subsequently tumor angiogenesis (Shih and Lindley, 2006). In 2004, bevacizumab was approved by the Food and Drug Administration (FDA) as part of combination therapy for metastatic colorectal cancers (Hurwitz et al., 2004). Since 2008, bevacizumab was approved for the treatment of non-small-cell lung, breast, kidney and ovarian cancers in combination with standard chemotherapy (Sandler et al., 2006; Harshman and Srinivas, 2010; Russo et al., 2017). The strong competition in the field led to the development of alternative strategies to inhibit angiogenesis. Since VEGF receptors possess a tyrosine kinase domain, several companies developed small ATP mimetics to inhibit the activity of tyrosine kinases receptors involved in angiogenesis (Qin et al., 2019). Another strategy was designed to inhibit the activity of mTOR, a kinase activated in response to the stimulation of receptors (Faes et al., 2017). The inhibitor molecules sorafenib (Nexavar^®^), sunitinib (Sutent^®^), everolimus (Afinitor^®^), temsirolimus (Torisel^®^), have for instance been extensively studied in several metastatic cancers (Ebos and Kerbel, 2011; Motzer et al., 2013; Patel et al., 2016; Faes et al., 2017). Sorafenib and sunitinib were the first multikinase inhibitors to be approved, in the therapeutic arsenal for metastatic renal cell carcinoma (RCC) and advanced hepatocellular carcinoma management on the base of increased progression free survival (PFS). However, the impact of these treatment on overall survival (OS) was limited (Yang et al., 2003; Sandler et al., 2006; Miller et al., 2007; Kerbel, 2008; Escudier et al., 2010). Moreover, they induced detrimental side effects such increased blood pressure and hand and foot syndrome (Motzer et al., 2013).

Renal cell carcinoma became a paradigm for the development of more efficient and less toxic agents. Hence, axitinib whose affinity for targets equivalent to those of sunitinib was higher, presented equivalent therapeutic effects with reduced toxicity (Motzer et al., 2013). New drugs were also developed for the treatment of RCC including pazopanib (Votrient^®^), vandetanib (Caprelsa^®^) or lenvatinib (Lenvima^®^) (Llovet et al., 2008; Escudier et al., 2014; Motzer et al., 2015; Rizzo and Porta, 2017). Lenvatinib exploited the inhibition of Fibroblast Growth Factor receptors that are key in endothelial cell proliferation.

Several tumor cells aberrantly expressed VEGFRs and exhibit exacerbated genetic plasticity following anti-angiogenic therapies that is highlighted by several mechanisms of adaptation/resistance. The crosstalk between tumor and stromal cells allows escape mechanisms counteracting the effects of anti-angiogenic therapies.

**FIGURE 2:**
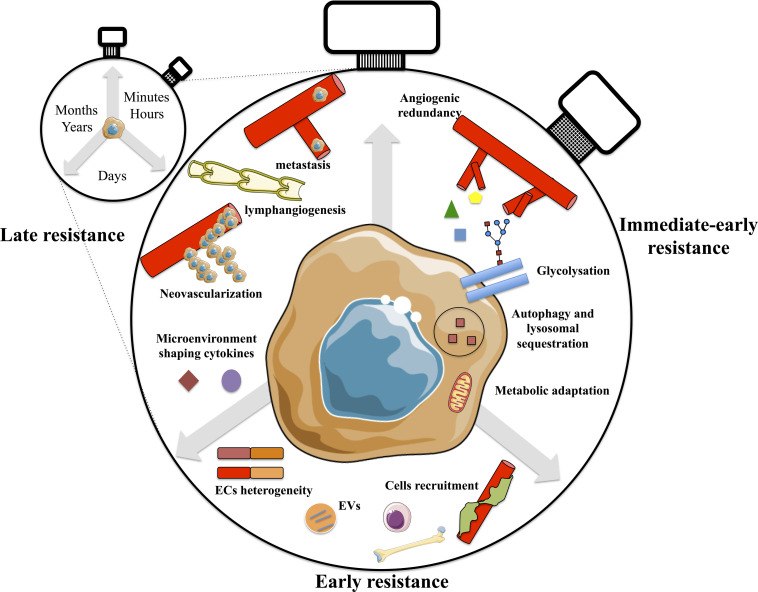
The adaptation to anti-angiogenic therapies; a clock ticking mechanism. Immediate-early resistance occuring within minutes to few hours following anti-angiogenic treatment involved angiogenic redundancy, glycosylation of VEGFR2, metabolic adaptation and sequestration of drugs in lysosomes inducing incomplete autophagy. Early resistance occurs during the following days after treatment and involved BMDCs, stromal cells recruitment into the tumor mass and ECs. The heterogeneity of ECs mediates resistance. Finally, within months following therapy, tumors adopt neovascularization strategies and increased lymphangiogenesis triggering metastasis and poor outcome in patients.

The objective of this review is to present an overview of the different resistance mechanisms to angiogenic therapies, from the earliest to the late ones, including tumor and stromal cells adaptation. The different mechanisms were divided into “immediate early” resistance mainly reffering to adaptation of tumor cells following exposure to the drug for few minutes/hours; into “early” resistance reffering to days/weeks after treatment exposure and into “late” one occuring several months/years after the treatment and depending on metastasis (Figure 2). Understanding the different spatiotemporal mechanisms leading to such resistance is essential to propose innovative therapeutic strategies for patients presenting innate or acquired resistances.

## “Immediate-Early” Resistance to Anti-Angiogenic

### Redundant Angiogenic Pathways and Hypoxia

Angiogenic redundancy is one of the earliest mechanisms leading to refractoriness or acquired resistance to anti-angiogenic therapies mainly targeting VEGFA and its receptors. Although VEGFA is the best known angiostimulatory protein, angiogenesis can be triggered by several growth factors including angiopoietins (ANGs), epidermal growth factor (EGFs), fibroblast growth factors (FGFs), hepatocyte growth factor (HGF), transforming growth factors (TGFs), placental growth factor (PlGF) or stromal cell-derived factor 1 (SDF1) (Xin et al., 2001; Bergers and Hanahan, 2008; Pardali et al., 2010; Brooks et al., 2012; Fagiani and Christofori, 2013). Except PlGF, which binds to VEGF receptors, all these angiogenic factors signal through different receptors expressed at the membrane of ECs (van Beijnum et al., 2015). This diversity of growth factors extents the toolbox of tumors to create blood vessels. Breast or pancreatic cancers for example rely on these angiogenic factors rather than on VEGFA and are poor responders to bevacizumab (Casanovas et al., 2005). Moreover, preclinical and clinical studies showed that anti-VEGFA antibodies and tyrosine-kinase inhibitors of VEGF receptors stimulate the production of these different growth factors (van Beijnum et al., 2015; Falcon et al., 2016; Haibe et al., 2020).

Preclinical studies for instance demonstrated an increase of SDF1 and PlGF in mice treated with anti-VEGFR2 compounds (Ebos et al., 2007; Fischer et al., 2007). The prolonged use of anti-VEGFR2 antibodies in transgenic mice models of pancreatic cancer stimulated the expression of ANG1 and FGFs. This increase correlated with a shorter survival (Casanovas et al., 2005). Comparable results were reported in head and neck squamous cell carcinoma (HNSCC) xenografts models. Indeed, microarray analysis, showed increased levels of FGF2 and its receptor (FGFR3) in bevacizumab-resistant tumors (Gyanchandani et al., 2013). These results were extended to lung cancer models resistant to angiogenesis-inhibitors, overexpressing EGFRs and FGFRs (Cascone et al., 2011). Clinical studies conducted on bevacizumab-treated colorectal cancer patients evidenced an increase of circulating PlGF, SDF1 and HGF levels (Willett et al., 2005; Kopetz et al., 2010). Equivalent results were obtained for glioblastoma patients treated with cediranib/Recentin^®^, a tyrosine kinase inhibitor of the VEGFR1, VEGFR2 and VEGFR3 (Batchelor et al., 2007, 2010). FGF and SDF1 increased expression was correlated with tumor relapse in cediranib-treated glioblastoma patients.

### Angiopoietin-2

Angiopoietins belong to a family of protein controlling vascular maturation during developmental and pathophysiological angiogenesis (Stratmann et al., 1998; Jeansson et al., 2011; Thurston and Daly, 2012). The predominant angiopoietins are ANG1 and ANG2. ANG1 mediates migration and survival of endothelial cells through binding to Tie2 receptor found on ECs of blood vessels and monocytes, whereas ANG2 promotes cell death and vascular regression (Hanahan, 1997; Maisonpierre et al., 1997). VEGFA and ANG2 promote neovascularization and ANG2 plays a key role in tumor relapse following anti-VEGFA treatment (Asahara et al., 1998). In preclinical models of anti-VEGFR-treated tumors, upregulation of ANG2 stimulates vascular remodeling and sprouting (Crawford and Ferrara, 2009). This observation was supported by clinical studies showing that patients suffering of colorectal cancers who are poor responders to bevacizumab, exhibit high serum levels of ANG2 (Ogawa et al., 2004; Goede et al., 2010). Equivalent results were obtained for melanoma and breast cancer patients treated with anti-angiogenic therapies. Increased serum level of ANG2 correlated with disease progression (Sfiligoi et al., 2003; Helfrich et al., 2009). Preclinical studies recently showed that simultaneous blockade of VEGFA and ANG2 inhibits angiogenesis and tumor growth (Brown et al., 2010; Kienast et al., 2013; Schmittnaegel et al., 2017; Wolf and Langmann, 2019). Clinical trials using such combination are ongoing for the treatment of metastatic colorectal cancers (NCT01688206, NCT02141295). Recently, the vanucizumab, a bispecific anti-ANG2/anti-VEGFA antibody has been evaluated in a phase I study. Vanucizumab displayed acceptable safety profile and encouraging anti-tumor activity (Hidalgo et al., 2018).

### Fibroblast-Growth Factors

Fibroblast-growth factors belongs to a family of 22 cell-signaling proteins involved in a broad variety of processes. FGF binds to tyrosine kinase receptors (FGFRs), expressed on tumor and stromal cells including endothelial cells, cancer-associated-fibroblasts or myeloid cells infiltrating tumors (Beenken and Mohammadi, 2009; Ornitz and Itoh, 2015). The FGF pathway promotes cancer progression and angiogenesis by activating RAS/RAF/MEK//ERK and PI3K/AKT/mTOR pathways (LaVallee et al., 1998; Hart et al., 2000). FGFs and FGFRs up-regulation, are involved in mechanisms of resistance to anti-VEGFA therapy (Casanovas et al., 2005; Kopetz et al., 2010). VEGFR2 inhibitors induce FGF2 expression and accelerate the growth murine pancreatic neuroendocrine tumors (Casanovas et al., 2005). Clinical studies on glioblastoma furthers confirmed this observation (Batchelor et al., 2007; Lee et al., 2019).

The proangiogenic role of FGF and its involvement in resistance to VEGFA inhibitors constitute a strong rationale for the development of inhibitors targeting the FGF and VEGFA pathways. The combined inhibition of FGF2 and VEGFA was highly efficient in preclinical models of head and neck carcinoma or pancreatic tumors (Casanovas et al., 2005; Gyanchandani et al., 2013). FGFR inhibitors notably restore the sensibility to bevacizumab in experimental models in mice suggesting a promising therapeutic combination (Gyanchandani et al., 2013). However, clinical investigations failed to demonstrate the relevance of this association (Norden et al., 2015; Semrad et al., 2017). Lenvatinib, a multiple receptor tyrosine kinase inhibiting the VEGFRs, FGFRs, and PDGFRs has shown promising therapeutic effects against various solid tumors and should be considered for counteracting resistance to anti-angiogenic agents (Suyama and Iwase, 2018).

### Plateled-Derived-Growth Factor

In the 1970’s several groups demonstrated the existence of growth factors for fibroblasts and smooth muscle cells derived from platelets (Paul et al., 1971; Bowen-Pope and Ross, 1982). These factors were named platelet-derived-growth factors (PDGF) and were one of the first growth factors to be characterized. By binding to their receptors PDGFRs, PDGFs are major mitogens for many cell types and actively participate in angiogenesis (Papadopoulos and Lennartsson, 2018). In cancer, PDGFs exert autocrine loops that stimulate tumor cell proliferation, and paracrine signaling for angiogenesis (Liu et al., 2011; Manzat Saplacan et al., 2017). Upregulation of PDGF was evidenced in glioblastoma patients following anti-angiogenic therapy (Liu T. et al., 2018). The blockade of PDGFR pathway increases the sensibility to VEGFA-neutralizing treatment, giving the rationale for new therapeutic opportunities. However, imatinib, a PDGFR inhibitor, in combination with bevacizumab, failed to demonstrate efficacy in renal cell carcinoma (RCC) patients (Hainsworth et al., 2007; Rock et al., 2007). Despite an increase of PFS, the VEGFRs and PDGFR inhibitor sunitinib is not curative for RCC patients (Motzer et al., 2009).

Based on the redundancy in angiogenic pathways, limited benefits to patients were observed by targeting a single angiogenic growth factor or its receptor. This redundancy is at the origin of innate or acquired resistance, by activation of alternative proliferation/survival pathways. Inhibition of ANG2-, FGF- or PlGF-mediated signaling pathways with those of VEGFA overcomes aspects of resistance to VEGFA blockade, but a sustained inhibition remains to be demonstrated.

### Transforming Growth Factor-β

The Transforming Growth factor-β (TGF-β) family regulates cell proliferation, differentiation and apoptosis (Massagué, 2000). In tumors, the role of TGF-β is ambivalent with tumor suppressive effects in early stage, thereafter switching toward tumor progression at later stages (Derynck et al., 2001). TGF-β induces the production of extracellular matrix and stimulates tube formation by ECs therefore inducing angiogenesis (Ferrari et al., 2009). Upregulation of TGF-β expression was reported in mice models of glioma resistant to anti-VEGF therapy (Park et al., 2016). Inhibition of TGF-β in hepatocellular carcinoma (HCC) and glioblastoma revealed anti-angiogenic benefit offering the rational to combine anti-TGF-β agents with anti-VEGF (Fransvea et al., 2009; Comunanza and Bussolino, 2017). The combination of galunisertib, a small inhibitor of TGF-β with sorafinib led to durable response in mice models of breast cancer (Holmgaard et al., 2018). TGF-β is also a major inducer of cancer associated fibroblast (CAF) development and fibrosis that are determinant in tumor aggressiveness. Targeting two hallmarks of cancer with one molecule probably explain the therapeutic response.

Combining anti-VEGF/VEGFR therapies to inhibitors of alternative angiogenic pathways appears relevant. However, the toxicity of such approach is an important issue. Treatment targeting concomitantly VEGFR and receptors involved in relapse is another option. One of the best example was the approval of cabozantinib (Cabometyx^®^) an inhibitor of VEGFR but also of cMET and AXL to actors involved in relapses after sunitinib treatment. Cabozantinib was approved as a second line treatment for RCC patient experiencing progression on sunitinib (Choueiri et al., 2016). It showed also a better efficacy as compared to sunitinib for RCC patients with poor or intermediate risk (Choueiri et al., 2017).

### Hypoxia, a Key Mediator of Angiogenic Redundancy

Hypoxia arises from the combination of high proliferative and metabolic rates with abberant tumor vascularisation with poor oxygen delivery (Semenza, 2014). Beside redundant pro-angiogenic pathways, tumor hypoxia is considered as an “immediate early” response to anti-angiogenic therapy. Although anti-angiogenic therapies reduce and normalize tumor vasculature, limiting tumor hypoxia, alternative theory defends an increased intra-tumor hypoxia (Kerbel and Folkman, 2002; Jain, 2005). Hypoxia plays an important role in resistance to conventional therapies leading to the selection of more aggressive stem cells and a shorter survival (Harris, 2002; Wilson and Hay, 2011; Chen et al., 2018). Indeed, anti-angiogenic agents induce intra-tumoral hypoxia and a concomitant stabilization of the hypoxia-inducible factors 1 and 2 alpha (HIF1/2α). HIF1 is considered as a tumor suppressor whereas HIF2 is considered as an oncogene. HIF1α is a major transcriptional regulator of angiogenic factors. It transactivates hundreds of pro-angiogenic genes, including growth factors (VEGFA, PlFG, FGF-2, PDGF) and their receptors (VEGFRs) (Hirota and Semenza, 2006; Rapisarda and Melillo, 2009). Moreover, HIF1 inhibits the production of anti-angiogenic factors, exacerbating angiogenesis (Hanahan and Folkman, 1996; Laderoute et al., 2000). Hence, HIF1 exerts also potent transcriptional inhibition especially following a long exposure in hypoxic conditions (Hantelys et al., 2019).

Hypoxia and HIF1 activation also trigger EMT and metastasis by regulating the expression of key genes such as *c-MET*, *CXCR4*, and lysyl oxidase (*LOX)*, events occurring later as discussed above (Joseph et al., 2018). Moreover, the hypoxic microenvironment generated following anti-angiogenic therapy stimulates β1-integrin expression, a well-known marker of resistance to cancer treatments (Foubert and Varner, 2012) which is consistent with its upregulation in clinical specimens of bevacizumab-resistant glioblastoma (Cordes and Park, 2007). Preclinical studies in mice models of glioblastoma demonstrated also the implication of β1-integrin in resistance to angiogenic therapies (Sidorov et al., 2016). The tumor microenvironment is hypoxic and the active metabolism of tumor cells induces the release of CO2 and lactate (Parks et al., 2013). The effect of hypoxia on tumor metabolism is detailled in the tumor metabolic adaptation part below.

The important role played by HIF in tumor aggressiveness stimulated the development of HIF inhibitors especially HIF2 that has oncogenic properties. Such treatments dissociate the HIF2α HIF1β dimer and consequently inhibit the transcriptional activation of HIF2. This treatment was successfully used in a multi-treated RCC patients (Chen W. et al., 2016). Hence, this treatment combined with classical anti-angiogenic drugs or immunotherapies (see below) is promising and should be further validated (Martínez-Sáez et al., 2017).

### Autophagy and Lysosomal Sequestration

Autophagy is a physiological process involving the sequestration of unnecessary or dysfunctional cell components and their degradation in lysosomes (Mizushima, 2007; Janku et al., 2011; David, 2012). In pathophysiological conditions, autophagy is an adaptative response to stress. In cancer, autophagy acts as a double-edged sword by serving as a pro-survival or pro-death process (Mathew et al., 2007). Autophagy plays an important role in enabling tumor cells to overcome harsh conditions arising from the microenvironment following treatment (Chandra et al., 2019). By enhancing the survival of tumor cells, it is indeed now considered as an important mechanism of resistance to cancer drugs (Li et al., 2017; Desantis et al., 2018). Hypoxia-induced autophagy favor the survival of hypoxic tumor cells (Brahimi-Horn et al., 2011). Two mechanisms drive hypoxia-dependent autophagy; the *non-selective* and the *selective* autophagy extensively reviewed (Chandra et al., 2019).

A cytoprotective role of autophagy was supported by several preclinical studies using radiation or imatinib as anti-cancer strategies (Miyazawa et al., 2010; Gewirtz, 2014). Resistance to sorafenib in hepatocellular carcinoma was attributed to increased activation of mTOR or Akt pathway triggering autophagy and cell survival (Zhai et al., 2014; Luan et al., 2019). The pro-tumoral role of autophagic processes in mediating resistance to anti-cancer treatments in HCC was highlighted by combining sorafenib to autophagy inhibitors (Shimizu et al., 2012; Lin et al., 2013; Hwang et al., 2015). These preclinical studies gave the proof of concept to initiate clinical trials combining inhibitors of autophagy to sorafenib.

In addition to tumor cells, stromal cells use autophagy as a mechanism of resistance to anti-angiogenic drugs. ECs, the direct targets on anti-angiogenic therapies, are inevitably exposed to drugs via the blood stream. Hence, resistance to sunitinib depends at least, on autophagy processes in ECs (Wu et al., 2020). Sunitinib-resistant RCC display an increased number of lysosomes allowing an enhanced sequestration of the drug which limits its therapeutic activity by isolating the drug from its cytoplasmic targets (Giuliano et al., 2015). The basic pKa of sunitinib induces its lysosomal sequestration., It prevents its accessibility to the tyrosine kinase domains of the receptors targeted by the drug (VEGFR1, 2, 3, PDGFR, CSF1R and cKIT), limiting the efficacy of the treatment.

### Tumor Metabolic Adaptation

The updated “Hallmarks of cancer: The Next Generation” includes the deregulation of cellular energetics as a key actor of tumor progression (Hanahan and Weinberg, 2011). Over the last decades, tumor hypoxia, by shaping cell metabolism was demonstrated as a key actor of tumor adaptation to anti-angiogenic therapies. Tumor cell metabolism and angiogenesis are tightly regulated by hypoxia (Semenza, 2014). Several genes involved in glycolysis are under HIF1 control, such as *GLUT1*, *GLUT3*, *PDK1* or *LDHA* (Favaro et al., 2011). The more hypoxic the cell, the more glycolysis is used, leading to pyruvate production. Instead of entering the tricarboxylic acid cycle, most of pyruvate is converted to lactate. This excess of lactate diffuses in the extracellular environment and is picked up by oxygenated cells, that revert the lactate to pyruvate and enhance their oxidative phosphorylation (Cassim et al., 2020; Parks et al., 2020). Consequently, their need for glucose decreased, and more glucose is available for the more hypoxic area of tumors (Nakajima and Van Houten, 2013). Following sunitinib treatment, the establishment of this symbiotic loop allows the proliferation of the remaining viable cells despite the dramatic increase of hypoxia following angiogenesis inhibition (Pisarsky et al., 2016).

In addition to low oxygen, increased acidification is also a hallmark of hypoxic tumors. It plays a key role in resistance to anti-cancer therapy (Erra Díaz et al., 2018). While mammalian cells protect their cytosol from acidification through expression of membrane transporters and exchangers such as the Na^+^/H^+^ exchanger (L’Allemain et al., 1985) and the monocarboxylate transporter 1 (Halestrap and Price, 1999), hypoxic tumors have developed additional mechanisms to regulate their pH. In solid tumors, the transcription of carbonic anhydrase (CA) IX is controled by HIF1. CAIX catalyzes the hydration of carbon dioxide (CO_2_) into H^+^ and bicarbonate (HCO3^–^) which is rapidly uptaken into cell by Na^+^-HCO3^–^ transporters sustaining alkaline pHi compatible with cell survival (Parks et al., 2013). In bevacizumab-resistant glioblastomas, increased levels of CAIX and of c-MET were observed (Jahangiri et al., 2013). Analysis of bevacizumab-resistant glioblastoma further revealed modifications in the expression of genes regulating cell metabolism, with (i) an increase of glycolysis-involved genes and (ii) a decrease of genes regulating oxidative phosphorylation (Kumar et al., 2013). Soluble CAIX is also correlated with a poor response to bevacizumab in breast cancers (Janning et al., 2019). Moreover, hypoxia leads to AMPK activation, inducing the metabolic switch from glycolysis to oxidative phosphorylation (McIntyre and Harris, 2015). Following anti-angiogenic therapy, tumor metabolism shifts from glycolysis to lipid consumption allowing tumor relapse (Sounni et al., 2014). Several clinical trials combining metabolism-targeting or hypoxia-targeting drugs with anti-angiogenics are ongoing (McIntyre and Harris, 2015). Recently, exciting novel concepts involving dual blockade of angiogenesis and metabolic adaptation have emerged and could revert the resistance to anti-angiogenic drugs (Jiménez-Valerio and Casanovas, 2017).

Recent findings demonstrated that metabolic reprogramming also occurs in TECs. TECs display upregulation of anabolic pathways in comparison to normal ECs. Unbiased meta-analysis revealed that *Aldh18a1* and *Sqle* were consistently induced in TECs raising the possibility to identify specific targetable TECs markers (Rohlenova et al., 2020).

### Glycosylation–Mediated Resistance

Activation of angiogenic receptors also occurs independently of ligand binding, therefore constituting another mechanism of insensitivity to cancer therapies. This process depends in part on galectins. They belong to a family of carbohydrate-binding proteins displaying high affinity for beta-galactoside (Camby et al., 2006). Galectin-1 is overexpressed in tumors and its expression correlated with metastatic dissemination and immune-escape (Hsu et al., 2013). Tumors refractory to anti-VEGFA treatments exhibit enhanced angiogenesis. Anti-VEGFA treatment and hypoxia increased galectin-1 production. Galectin-1 binds to N-glycans glycoproteins on endothelial cells, including VEGFR2. This binding prolongs the presence of VEGFR2 at the cell surface and promotes angiogenesis without VEGFA binding (Croci et al., 2014; Stanley, 2014). Further clinical investigations are needed to consider galectins as relevant targets for anti-angiogenic therapies.

### Matrix Metalloproteinases

Matrix metalloproteinases (MMPs) play a key role in angiogenesis and in tumor progression (Deryugina and Quigley, 2010). MMPs can be pro- or anti-angiogenic depending of their categories. On the one hand, MMP-3 and MMP-7 display anti-angiogenic properties (Deryugina and Quigley, 2010). On the other hand, MMP-2 and MMP-9 promote the release of VEGFA from the ECM sustaining angiogenesis (Bergers et al., 2000). MMP-1 induces matrix remodeling and migration of ECs (Chun et al., 2004). Hence, it is now established that MMP inhibitors can induce tumor progression by favoring tumor angiogenesis. Therefore, MMP inhibitors combined to inhibitors of angiogenesis should be considered as a therapeutic option (Winer et al., 2018).

### Tumor Stroma

Tumors are a complex association of cancer cells as well as a stromal compartment with cellular and noncellular components. Tumor stroma plays crucial roles in tumor progression and in resistance to treatments. The dense tumor stroma can limit the access of therapeutic agents to their target due to fibrosis, high interstitial pressure and degradation of drugs by stromal enzymes (Valkenburg et al., 2018). The rigid extracellular matrix can reduce blood vessel density, creating a barrier that drugs cannot perfuse (Olive et al., 2009). In parallell, the high interstitial pressure in tumor microenvironment affects drug delivery (Provenzano and Hingorani, 2013). Beside these effects, the cytochrome P450, expressed by fibroblasts, metabolizes toxic molecules including therapeutic drugs contributing to agressive behaviors of tumors (Hirth et al., 2000). In mice models of lung cancer, treatment with bevacizumab led to acquired resistance via upregulation of VEGFA, FGF2 and its receptor FGFR2 and PDGFR in stromal cells (Mitsuhashi et al., 2015). It now becomes evident that cancer therapies should include strategies to target and constrain the tumor stroma. Some agents targeting CXCR4, TGF-β or hyaluronic acid are currently under clinical consideration (Valkenburg et al., 2018).

## Early Resistance to Anti-Angiogenic Therapies

### Recruitment of Local Stromal Cells

Cells constituting the tumor environment play a key role in the resistance to angiogenesis inhibitors, especially cancer-associated fibroblasts (CAFs) and pericytes.

#### Cancer-Associated Fibroblasts

Cancer-associated fibroblasts are the principal component of the stroma within the tumor microenvironment. They exhibit diverse functions including matrix remodeling, crosstalk with tumor, endothelial or immune cells, promoting tumorigenesis. CAFs notably allow the recruitment of endothelial progenitors cells (EPCs) and bone-marrow-derived cells (BMDCs) through SDF1 expression and stimulation of its receptor CXCR4 on EPCs (Orimo et al., 2005). The role of EPCs and BMDCs are discussed in the next part. CAFs also promotes angiogenesis through the expression of galectin-1, VEGFA, FGF, HGF or PDGF (Tang et al., 2016; Wang et al., 2019). In tumor cells deficient for VEGFA, CAFs produce VEGFA to sustain angiogenic processes (Dong et al., 2004). CAFs isolated from anti-VEGFA resistant tumors, exhibit high levels of ANG2, and PDGF promoting tumor growth (Crawford and Ferrara, 2009). The pro-angiogenic and pro-invasive role of CAFs in resistance to antiangiogenic drugs can also arise from metalloproteinases (MMPs) production (Sternlicht et al., 1999; Boire et al., 2005).

Blocking the pro-angiogenic role of CAFs with an anti-FGF-2 ^125^I-radiolabeled antibody resulted in the inhibition of HCC tumor growth and decreased angiogenesis (Wang et al., 2012; Hu et al., 2016). Lenvatinib (Lenvima^®^), which inhibits VEGFRs has a potent anti-angiogenic effect and inhibits also FGF receptors involved in anti-angiogenic resistance. It is now used in the therapeutic arsenal against kidney tumors (Motzer et al., 2016). Brivanib from Bristol Myers Squibb, an anti-VEGFR and FGFR, increased the PFS of 43 patients with recurrent endometrial cancers in a phase II clinical trial (Powell et al., 2014; Hosaka et al., 2018).

#### Pericytes

Blood vessels are composed of two interacting cell types: the ECs, forming the inner face of vessels, and perivascular cells, called pericytes. Pericytes are peri-endothelial cells that directly interact with ECs, regulating vessel diameter, permeability and therefore the blood flow (Bergers and Song, 2005). Recruitment of pericytes by ECs relies, at least, on the PDGF-PDGFR signaling (Abramsson et al., 2003). Pericytes negatively regulate the proliferation of ECs promoting maturation of neo-vessels (Orlidge and D’Amore, 1987). In preclinical models of glioma or RCC, an increased tumor blood vessel coverage by pericytes following sunitinib or bevacizumab treatments was observed (Norden et al., 2009; Cao et al., 2013; Pinto et al., 2016). Residual tumor vessels, in a preclinical model of colorectal cancer, were heavily covered by pericytes following treatment with Anti-ANG2 antibodies (Thomas et al., 2013). Moreover, the number of vessels covered by pericytes following sunitinib was correlated to aggressiveness of RCC (Cao et al., 2013). Pericyte coverage enhances tumor resistance to these therapies through limited ECs proliferation and through the availability of survival signals (Orlidge and D’Amore, 1987). These different mechanisms highlight the role of pericytes in the resistance to anti-angiogenic treatments observed in the clinic. Therefore, inhibiting blood vessels maturation by targeting blood vessel coverage by pericytes is a relevant strategy to overcome the resistance to anti-angiogenic therapies. Inhibition of PDGFR by imatinib and sunitinib in combination with anti-VEGFR showed anti-tumor effects on experimental tumors in mice (Pietras and Hanahan, 2005). FGF2/FGFR2 signaling and PDGF/PDGFR signaling crosstalk to enhance pericyte proliferation and recruitment (Hosaka et al., 2018). PDGF stimulates the pericytes-fibroblast transition, which contributes to metastatic processes (Hosaka et al., 2016). Therefore, inhibition of PDGF-mediated recruitment of pericytes showed potent anti-tumor effects (Thijssen et al., 2018). Hence, disrupting pericytes support, by using an anti-PDGFR and destabilizing pre-existing tumor vasculature with an anti-VEGFR, is an attractive strategy to overcome tumor refractoriness to conventional anti-angiogenic therapies.

### Recruitment of Bone-Marrow Derived-Cells (BMDCS)

Anti-angiogenic therapies normalize vessels but also increase intra-tumoral hypoxia leading to the recruitment of bone marrow-derived cells (BMDCs) (Jain and Duda, 2003). Infiltration of BMDCs in tumors has been linked to tumor progression and angiogenesis for several years (Jain and Duda, 2003). As above-mentioned, anti-angiogenic therapies stimulate the production of pro-angiogenic factors (VEGFA, Angiopoietins, FGFs). However, the stress induced by the treatment stimulates inflammatory pathways involved in the production of cytokines such as SDF1, IL-8 or granulocyte colony-stimulating factor (G-CSF). These cytokines trigger the recruitment of BMDCs that exhibit high plasticity and pro-angiogenic potential limiting the efficacy of anti-angiogenic drugs (van Beijnum et al., 2015).

#### CD11b^+^ Gr1^+^ Myeloid-Derived Suppressor Cells

Myeloid-derived suppressor cells (MDSCs), also known as CD11b^+^ Gr1^+^ cells are composed of a mixed population including neutrophils, macrophages and dendritic cells displaying immunosuppressive and pro-tumorigenic capacities (Yang et al., 2004; Marigo et al., 2008; Crawford and Ferrara, 2009). Preclinical and clinical studies evidenced an increased number of MDSCs in cancers, promoting tumorigenesis and angiogenesis (Yang et al., 2004; Serafini et al., 2006; Diaz-Montero et al., 2009). The infiltration of tumors by MDSCs is therefore correlated with a poor outcome in patients. It participates in mechanisms of resistance to anti-angiogenic therapies (Shojaei and Ferrara, 2008). Indeed, tumors resistant to anti-VEGFA-treatments presented increased infiltration of MDSCs in comparison to anti-VEGFA sensitive tumors (Shojaei et al., 2007a). The presence of tumor infiltrating Th-17 cells induces the expression of G-CSF by CAFs and increased production of IL-6 and SDF1 by CAFs and tumor cells, allow the recruitment of MDSCs (Shojaei and Ferrara, 2008; Shojaei et al., 2009). Upregulation of G-CSF by resistant tumors triggers prokinectin-2 (Bv8) overexpression in the bone marrow (BM). Bv8 induces the migration of progenitor cells from the BM to the tumor. Anti-Bv8 antibodies reduce MDSCs recruitment and inhibit tumor growth and angiogenesis, suggesting a role of Bv8 in relapses following anti-VEGFA treatment (Shojaei et al., 2007a, b). Moreover, hypoxia induces resistance to sunitinib in glioblastoma, breast and metastatic RCC by increasing the recruitment of MDSCs to the tumor niche (Finke et al., 2011; Piao et al., 2012). In agreement with these observations, depletion of MDSCs sensitized tumors to anti-angiogenic therapies, highlighting their pivotal role in resistance (Holmgaard et al., 2016).

Among the MDSCs population, increased tumor-infiltration by neutrophils promote resistance to bevacizumab. Neutrophils induce Bv8-dependent tumor angiogenesis independently from the VEGFA signaling. Preclinical studies demonstrated that blockade of Bv8 decreases the recruitment of MDSC and angiogenesis (Shojaei et al., 2007b; Piao et al., 2012).

Macrophages, specialized phagocytic cells, also display plasticity and shape their phenotype in response to environmental conditions, making them a relevant candidate for treatment resistance (Ruffell and Coussens, 2015; Sarode et al., 2020). The first relationship between macrophages and angiogenesis was proposed in Knighton et al. (1983). Depending on their localization and on their polarization profiles, macrophages are pro- or anti-tumoral actors (Cheng et al., 2019). Recruitment of macrophages in tumors is induced by several cytokines including VEGFA or M-CSF. Macrophages secrete growth factors such as VEGFA or EGF triggering angiogenesis (van Beijnum et al., 2015). They also secrete matrix metalloproteases, and physically associate with ECs, promoting angiogenesis. In several preclinical studies, anti-VEGFA therapies reduced macrophage infiltration (Salnikov et al., 2006; Dineen et al., 2008). Nevertheless, specific macrophages with immunoglobin-like and EGF-like domains, the Tie-2-expressing macrophages (TEM), are recruited in hypoxic zones and by ANG2 (Murdoch et al., 2007). TEM also promote angiogenesis and tumor progression in hypoxic environment, through upregulation of HIF1α (De Palma and Naldini, 2011). Therefore, macrophages contribute to anti-angiogenic resistance. Although ANG2 inhibitors do not prevent the recruitment of TEM, they decrease their activity, illustrated by a downregulation of growth factors production and a decrease of their physical association with blood vessels (Mazzieri et al., 2011).

These results suggest that BMDCs are therapeutic targets for counteracting tumor refractoriness to anti-angiogenic therapies. Inhibition of the SDF1 pathway notably prevents BMDCs tumor infiltration and overcomes such resistance (Liu et al., 2010). Equivalent results were obtained with anti-Bv8 antibodies (Hasnis et al., 2014). Clinical studies recently demonstrated that plasma TEM are predictive markers of anti-angiogenic treatment failure in colorectal and ovarian cancers (Jayson et al., 2018). However, clinical investigations consisting in preventing tumor-infiltration of TEM are needed to further consider this therapeutic perspective.

#### Endothelial Progenitors Cells

The discovery of endothelial progenitor cells (EPCs) in adults and their putative vascular-promoting properties has generated debate in the field of vascular biology (Pasquier and Dias, 2010). EPCs were first isolated in 1997 by Asahara et al. (1997) EPCs are subtypes of stem cells that originate from the bone marrow. A controversy concerning their origin, their isolation and their functioning still exists. EPCs have high proliferative potential, capable of differentiation into mature ECs, therefore contributing to neovascularization and angiogenesis (Asahara et al., 1997; Reale et al., 2016). Several surface markers (CD133, CD34, and VEGFR2) characterize bone marrow derived-EPCs. They acquired CD31 and CD146 expression during their transport to the blood. They become mature ECs in the target tissues where they expressed VEGFR2, CD31, CD136, VE-cadherin, eNOS and von Willebrand factor (Puente et al., 2013). EPCs have a dual role in promoting angiogenesis into the tumor tissue; they regulate the angiogenic process through the production of growth factors and provide structural function in sprouting nascent vessels (Puente et al., 2013). The main chemo-attractants for EPCs in tumor tissue are VEGFA and SDF1, released by ECs, cancer cells and CAFs (Orimo et al., 2005; Grunewald et al., 2006). When recruited, EPCs promote angiogenesis by differentiating in ECs and by incorporating newly formed blood vessels (Puente et al., 2013). Anti-angiogenics, through hypoxia and HIF1α activation lead to the production of VEGFA and SDF1 by tumor cells triggering mobilization and recruitment of EPCs (Ceradini et al., 2004). Activated EPCs secrete pro-angiogenic factors leading to limited effects of anti-angiogenic therapies. Although the precise mechanism of EPCs-induced neovascularization remains poorly understood, recent studies in non-small-cell lung carcinoma (NSCLC) demonstrated a key role of histone deacetylase 7 (HDAC7) in the regulation of angiogenic genes (Wei et al., 2018). Nevertheless, the therapeutic implication of EPCs still remains to be elucitated.

#### Heterogeneity of Tumor Endothelial Cells

Heterogeneity of tumor endothelial cells (TECs) contributes to resistance to anti-angiogenic therapy (Maishi et al., 2019). TECs cover the inner surfaces of tumor blood vessels and are consequently directly exposed to anti-angiogenic drugs. TECs differ in several points from normal ECs. They display cytogenetic abnormalities, upregulation of pro-angiogenic factors and expression of stemness genes leading to drug resistance (Hida et al., 2004; Maishi et al., 2019). TECs express high levels of VEGFR1, VEGFR2, VEGFR3, and Tie-2 leading to strong responses to their respective angiogenic ligands (Alessandri et al., 1999; Bussolati et al., 2003). Moreover, TECs produce nonconventional growth factors such as biglycan, LOX and pentraxin, sustaining angiogenesis processes (Maishi et al., 2019). These observations led to the development of LOX inhibitors. Inhibition of LOX and biglycan reduces tumor metastasis suggesting the relevance of LOX targeting (Yamamoto et al., 2012; Osawa et al., 2013).

The hypoxic tumor microenvironment stimulates the expression of stemness genes in TECs, such as stem cell antigen 1 (Sca-1), MDR-1 and aldehyde lactate deshydrogenase (ALDH), leading to resistance to paclitaxel and to fluorouracil (5-FU) (Xiong et al., 2009). The vascular stem cells, that constitute a minor population in tumors, were suggested to contribute to tumor resistance to conventional chemotherapy and to anti-angiogenic treatments. Indeed, TECs derived from HCC are also more resistant to sorafenib, in comparison to human umbilical vein endothelial cells (HuVECs) (Xiong et al., 2009).

Transforming growth factors can originate from dedifferentiation of tumor cells, monocytes or from EPCs contributing to high heterogeneity and to resistance to anti-angiogenic treatments. Although cancer cells acquired drug resistance is well documented, the heterogeneity of TECs must be considered as a major actor. Recent single cell RNA-sequencing studies revealed endothelial cell heterogeneity following anti-VEGF therapy (Zhao et al., 2018). TECs can be classified into tip-like, transition and stalk-like cells. The sequencing of 56,771 endothelial cells from human/mouse (peri)-tumoral lung cells revealed different phenotypes following anti-angiogenic treatment. Tip-like signatures correlated with patient survival and tip-like TECs were most sensitive to anti-VEGF therapies (Goveia et al., 2020).

Among TECs-targeting therapies, inhibitors of CXCR4 were scrutinized since TECs are CXCR4-enriched populations is associated with a poor outcome in HCC. Inhibition of CXCR4 induces promising anti-tumor response mainly by preventing recruitment of BMDCs in the tumor mass and must be considered as a future therapeutic option (Kioi et al., 2010).

#### Extracellular Vesicles

Metastatic dissemination of cancer cells relies on several parameters and notably on the bi-directional communication between primary tumor and future metastatic tissues. This crosstalk essentially involves the production of particles by cancer or stromal cells. These particles are known as Extracellular vesicles (EVs). EVs carry onco peptides, RNA species or lipids from donor to recipient cells, triggering phenotypic changes of the future pre-metastatic niches (Xu et al., 2018). EV stimulate angiogenesis by transporting growth factors (VEGFA, PDGF, FGF-2), transcription factors (STAT3 and STAT5) or micro-RNAs (Todorova et al., 2017).

Recently, the emergence of EVs as a novel player of drug resistance has gained interest. EVs transfer drugs from resistant to sensitive cells triggering cell resistance (Maacha et al., 2019). VEGFA contained in EVs correlates with disease progression in bevacizumab treated patients, raising the possibility that resistance to bevacizumab relies on this process (Ko et al., 2019). Moreover, bevacizumab could be shed and exported by EVs leading to therapeutic escape (Simon et al., 2018).

## Late Resistance

### The Angiogenic-Dormancy as an Intrinsic Resistance Mechanism

Metastases can remain for months or years in a quiescent, dormant state, in the tissue they colonized. These micro-metastases constitute a residual disease characterized by the persistence of tumor cells, undetectable by conventional diagnostic techniques. The tumor dormancy can be defined as the lag in tumor growth occurring between primary tumor formation and the appearance of clinically detectable metastases (Yadav et al., 2018). The presence of disseminated tumor cells (DTCs) in bone marrow of prostate-cancer and breast-cancer patients have been reported before the development of overt metastases (Banys et al., 2012; Lam et al., 2014). Three molecular mechanisms characterize tumor dormancy: mitotic arrest, immunological and angiogenic dormancy (Senft and Ronai, 2016). The angiogenic dormancy may explain the reasons why angiogenic therapies simply delay tumor progression. More than 20 years ago, pharmacological inhibition of angiogenesis was found to induce dormancy in several mouse models (Holmgren et al., 1995; O’Reilly et al., 1996). The supposed but unproven “angiogenic switch” is supposed to play a key role in the maintenance of the dormancy, since dormant cells upregulate angiogenesis inhibitors such as thrombospondin-1 (TSP-1) (Senft and Ronai, 2016). Despite the lack of clinical evidences, the “angiogenic switch” of dormant cells has to be considered in cancer relapse following treatment arrest.

### Induction of Cancer Stem Cells

Cancer stem cells (CSCs) constitute a small population of cells within tumor exhibiting abilities of self-renewal, differentiation and high tumorigenicity potential. They play a key role in the initiation of cancer and in the metastatic cascade. In 2003, CSCs were first identified in human breast and brain cancers (Al-Hajj et al., 2003; Singh et al., 2003). CSCs express CD44, CD24, CD29, CD90, CD133 and aldehyde deshydrogenase (ALDH1) allowing their identification (Yu et al., 2012). CSCs drive angiogenesis in hypoxia and HIF mediates CSCs proliferation and self-renewal (Tong et al., 2018). CSCs was suggested to give rise to endothelial cells and thus neovascularization processes (Fujita and Akita, 2017). Moreover, CSCs can differentiate in pericytes, supporting tumor vessel function (Cheng et al., 2013).

Their tumor initiating properties and their metastatic potential suggest that CSCs are involved in resistance to therapies. Conventional treatments including chemo- and radiation therapies generate the production of CSCs promoting tumor escape (Chen X. et al., 2016; Li et al., 2016; Liu L. et al., 2018). CSCs are actors of anti-angiogenic resistance. Preclinical studies on experimental models of breast cancers showed that sunitinib and bevacizumab increase the CSCs populations through HIF1 activation (Conley et al., 2012). These results indicate that administration of anti-angiogenic agents accelerate tumor growth by increasing CSCs population. Several CSCs-targeting therapies are currently under development. Inhibition of ALDH1 prevents CSCs enrichment and reduces tumor formation of experimental triple-negative breast cancer and NSCLC in mice (Schech et al., 2015; MacDonagh et al., 2017). Evaluation of CD44, CD133 or Hedgehog inhibitors are currently under considerations for further clinical developments (Shibata and Hoque, 2019).

### Induction of Epithelial-Mesenchymal Transition and Invasion

Epithelial-mesenchymal transition (EMT) defines the acquisition of characteristics of invasive mesenchymal cells by epithelial cells. EMT is implicated in tumor invasion and metastasis and correlates with poor clinical outcome in several solid tumors (Mittal, 2018). During the EMT process, epithelial cells lose their phenotypes, with a downregulation of E-cadherin and α-catenin and acquire mesenchymal markers (N-cadherin, vimentin, fibronectin) leading to cell mobility and invasiveness (Zeisberg and Neilson, 2009). Several signaling pathways induce EMT (TGF-β, Wnt, Notch), by controlling the transcription factors Snail, Slug, ZEB1/2 and Twist (Garg, 2013). Hypoxia and HIF1α are also well-known drivers of EMT. The expression of Twist and Snail, the downregulation of E-cadherin and the induction of vimentin promoting tumor invasiveness, have been reported following anti-angiogenic treatments (Cooke et al., 2012; Maione et al., 2012). Similarly, enhanced invasiveness and growth capacity of glioblastoma and RCC cells have been demonstrated following VEGFA inhibition (Grepin et al., 2012; Lu et al., 2012). This enhanced invasion abilities led to metastatic dissemination, a later step discussed in part 3.

Several studies highlighted the role of the tyrosine kinase receptor c-MET in promoting tumor invasiveness and metastasis in response to anti-angiogenic therapies (Pàez-Ribes et al., 2009; Lu et al., 2012; Sennino et al., 2012).

Although sunitinib and anti-VEGFA decreased tumor volume, invasiveness, hypoxia and EMT markers are increased (Ebos et al., 2009; Mizumoto et al., 2015). In addition, c-MET and the phosphorylated active forms of c-MET also increased as a consequence of treatment-induced hypoxia. The c-MET pathway is one of the most investigated pathways in the field of resistance to anti-angiogenic therapies. Its stimulation through HGF binding, triggers the activation of the RAS/RAF/MEK/ERK, PI3K/AKT/mTOR, and STAT3 pathways promoting tumor growth and invasiveness (Jeon and Lee, 2017). Bevacizumab-treated glioblastoma patients have increased relapse in comparison to bevacizumab-untreated patients. This clinical observation was recently linked to the upregulation of c-MET and phospho-c-MET (Jahangiri et al., 2013). Hence, c-MET is a robust actor of anti-angiogenic resistance by promoting EMT-like phenotype and invasiveness in glioblastoma. This observation has subsequently led to development of c-MET inhibitors. Cabozantinib, a promising multi-kinase inhibitor of c-MET, VEGFR2, and AXL, improves overall survival of RCC patients with bone metastases (Motzer et al., 2018).

### Lymphangiogenesis Induction

Historically, lymphatic vessels were considered as passive participants in metastatic dissemination, only acting as channels for tumor cells transit. Nowadays, it becomes evident that lymphatic vessels have an active role in promoting metastasis. The first pro-lymphangiogenic factors identified more than 20 years ago were the VEGFC and VEGFD that bind to VEGFR3 expressed on lymphatic endothelial cells triggering lymphangiogenesis (Joukov et al., 1996; Yamada et al., 1997). Overexpression of VEGFC and VEGFD increases the number of tumor-associated lymphatic vessels and the incidence of lymph node metastases (Christiansen and Detmar, 2011). Moreover, overexpression of VEGFC and VEGFD is correlated to intra-tumoral lymphatic vessel density, lymph node metastasis and poor outcome in patients with melanoma and breast cancers (Mohammed et al., 2007; van der Schaft et al., 2007). More recently, HGF, c-MET, Tie-2, PDGF and FGF were also identified as pro-lymphangiogenic factors (Christiansen and Detmar, 2011). Immunohistochemical analysis of tumor samples showed that lymphatic vessel invasion (LVI) correlated with lymph node metastasis (Christiansen and Detmar, 2011). Moreover, tumor cells through the expression of chemokine receptors exploit the lymphatic network to form metastases. Indeed, CXCR4 and CCR7 expressed on human breast cancer cells promote metastasis to organs expressing their respective ligands, SDF1 and CCL21 (Müller et al., 2001). CXCR4 is upregulated by hypoxia. Since dissemination to distant organs is governed by the SDF1 gradient, CXCR4/SDF1 antagonists inhibited lymph nodes spreading of cancer cells in experimental tumors in mice (Müller et al., 2001).

Drugs destroying blood vessels stimulate the development of tumor lymphatic vessels contributing to treatment failure. Tumors from sunitinib-treated RCC patients in a neoadjuvant setting exhibit increased lymphatic vessels and increased lymph node invasion. This detrimental effect is explained at least by the stimulation of VEGFC expression following sunitinib administration (Dufies et al., 2017a). Indeed, sunitinib stimulate *vegfc* gene transcription, mRNA stability and protein production and the subsequent VEGFC-dependent development of lymphatic vessels. Moreover, hypoxia upregulated VEGFC expression (Morfoisse et al., 2014; Ndiaye et al., 2019). Lymphangiogenesis participates in treatment failure and its targeting can be considered in the therapeutic arsenal but only for advanced tumors.

### Microenvironment Shaping by Cytokines

The central role played by VEGFA plus ELR+CXCL cytokines and especially CXCXL8/IL-8 was first documented by Sparmann and Bar-Sagi (2004) in colon cancers. The role of ELR+CXCL and their receptors-CXCR1/2 on tumor cell proliferation, angiogenesis and microenvironment adaptation following anti-angiogenic therapies was highly documented (Vandercappellen et al., 2008). The pro-inflammatory interleukin (IL-1 β stimulates CXCL7 production in RCC models resulting in tumor growth (Grepin et al., 2012; Grépin et al., 2014). CXCL7 is a predictive marker of sunitinib efficacy in RCC (Dufies et al., 2017b). CXCL5 in response to lysosomal sequestration of anti-angiogenic drugs plays also a key role in resistance to anti-angiogenic in renal and breast cancers (Giuliano et al., 2019). Inhibitors of CXCR1 and CXCR2 efficiently inhibit the growth of experimental HNSCC and RCC by decreasing tumor cell proliferation, angiogenesis and inflammation (Dufies et al., 2019).

### Novel Neovascularization Modalities

Beside angiogenesis, new vascular networks are generated by the attraction of endothelial progenitor cells, intussusseptive angiogenesis, vessel co-option and vasculogenic mimicry.

#### Vessel Co-option

Tumor can use alternative ways to obtain blood supply, and therefore counteracting the effects of anti-angiogenic therapies. Tumor cells can hijack pre-existing blood vessels of the surrounding non-tumoral tissue and migrate along these vessels. This process, which occurs in the absence of angiogenic growth factors, is called vessel co-option (Kuczynski et al., 2019). Basically, the cancer cells migrate along the surface of pre-existing vessels leading to their incorporation in the tumor mass. Vessel co-option has been extensively reported in histopathological specimens of lung, liver and brain cancers (Nakashima et al., 1995; Pezzella et al., 1997; Offersen et al., 2001; Winkler et al., 2009; Yao et al., 2018). This process sustains the growth of brain metastases emerging from melanomas, liver and breast cancers (Leenders et al., 2004; Kuczynski et al., 2016, 2019).

A major question is whether vascular co-option constitutes an intrinsic resistance or does it occur as an acquired resistance mechanism following therapy. Inhibition of VEGFA promotes cancer invasion, inducing vessel co-option *in vivo*. Mechanistic studies identified the actin-related protein, Arp2/3, c-MET, ZEB2- and WNT- EMT dependent signaling as promoters of cell motility and vessel co-option (Navis et al., 2013; Depner et al., 2016; Frentzas et al., 2016). Simultaneous blockade of VEGFA and ARP2/3, VEGFA and c-MET or VEGFA and ZEB2 suppresses tumor invasion (Sennino et al., 2012; Depner et al., 2016; Frentzas et al., 2016). Other therapeutic approaches include the blockade of cell-adhesion receptors, since tumor cells adhere to endothelial cells during co-option. Hence, a β1-integrin inhibitor combined with bevacizumab induced sustained anti-tumor response in bevacizumab-resistant glioma xenografts (Carbonell et al., 2013; Jahangiri et al., 2014).

The prognostic value of vessel co-option in cancer patients remains to be elucidated. Bevacizumab-treated colorectal cancer patients with liver metastases demonstrated a limited response due to vessel co-option (Frentzas et al., 2016). Combining cell-motility or cell-adhesion inhibitors with anti-angiogenic compounds deserves to be considered as a therapeutic alternative.

#### Vasculo Mimicry

The vasculo-mimicry is defined as the formation of vascular-like structures by non-vascular cells. In 1999, it was first reported that tumor can dedifferentiate and form vascular-like structure (Maniotis et al., 1999). Later, vasculo mimicry has been described in several tumor types such as breast, ovarian cancers or Ewing sarcoma (Sood et al., 2001; Shirakawa et al., 2002; van der Schaft et al., 2005). This dedifferentiation is accompanied by the acquisition of endothelial features such as VE-cadherin or Tie-2 expression (Maniotis et al., 1999). In addition, HIF1α is an important regulator in the process of vasculo-mimicry (Delgado-Bellido et al., 2017). Despite this dedifferentiation, tumors remain refractory to anti-angiogenic therapy (van der Schaft et al., 2004). Bevacizumab elicits vasculo-mimicry of tumors leading to tumor escape and metastasis (Xu et al., 2012). Sunitinib stimulates vasculo-mimicry by differentiating tumor cells to endothelial-like cells (Serova et al., 2016; Sun et al., 2017). Nevertheless, further studies are needed to clarify the correlation between vasculo-mimicry and resistance to anti-angiogenic therapies.

### Increased Metastasis Rate

The ultimate consequence of the resistance of anti-angiogenic therapies is the increased rate of metastasis. As developed in the previous parts, anti-angiogenesis therapies lead to (i) intrinsic reprogramming of tumor cells with upregulation of alternative pro-angiogenic pathways, increased of lymphangiogenesis-related genes and processes and initiation of EMT (ii) conditioning the microenvironment, with the recruitment of local- and bone marrow-derived cells or used novel neovascularization modalities. All of these mechanisms lead to increased metastatic rate. Ten years ago, Ebos et al. (2009); Pàez-Ribes et al. (2009) were the first to describe the association between anti-angiogenesis drugs and increased distant metastases. Preclinical models of breast cancers showed that sunitinib enhance lung and liver metastasis (Ebos et al., 2009). Anti-angiogenic treatments can make the host more permissive for metastatic seeding. Sunitinib-treated mice exhibit vascular changes such as reduced pericyte coverage and increased leakiness of normal vessels (Chung et al., 2012; Maione et al., 2012; Singh et al., 2012). Therefore, these systemic actions facilitate the creation of a metastatic niche at distance from the primary tumor.

Increased metastasis rate following anti-angiogenic therapies are highly variable and depends on several parameters such as the type of treatment, the dose and the schedule. Singh et al. (2012) showed that sunitinib enhanced the agressiveness of tumor cells whereas the use of an anti-VEGF antibody did not. Chung et al. (2012) further demonstrated that inhibition of VEGF signaling by antibodies does not promote metastasis, in contrast to small molecule RTK inhibitors at elevated-therapeutic drug dosages. Dosing and scheduling of anti-angiogenic administration can also induce resistance. Short-term and high dose of sunitinib increased growth of breast cancer and enhance liver and lung metastasis (Ebos et al., 2009). In contrast, treatment with low dose of sunitinib did not induce metastasis (Welti et al., 2012).

## Conclusion and Future of Anti-Angiogenic Therapies

Angiogenesis processes, through the establishment of a new vascular network, are an important contributor to tumor development and metastatic dissemination. Once the tumor has reached 1-2 mm^2^, the core of tumors become hypoxic and tumor cells counteract hypoxia by the production of angiogenic growth factors. Among them, VEGFA is one of the most important. Targeting the VEGFA/VEGFRs represented a great breakthrough in the therapeutic management of cancer patients. Unfortunately, complete responses are rare, and tumors counteract this inhibition through different processes. The molecular mechanisms of resistance are not fully understood and deciphering them has gained interest. It is now evident that several mechanisms exist. They involve a wide range of processes; (i) the earliest, with the upregulation of genes involved in angiogenic redundancy, EMT or the lysosomal sequestration of drugs, to the latest (ii) with an adaptation of the tumor microenvironment, reflected by the recruitment of progenitors cells, lymphangiogenesis, and adapted neovascularization modalities. All these mechanisms allow tumor metastasis and serve as limitations to anti-angiogenic drug efficacy.

Hence, combining treatments targeting tumor cells and cells of the tumor microenvironment should limit resistance and should improve patients’ survival. One of the first and of the obvious way of resistance involves angiogenic redundancy by multiple growth factors as suggested by the anti-tumoral effects of FGF inhibition and bevacizumab (Casanovas et al., 2005; Gyanchandani et al., 2013). Although combining anti-angiogenic therapies may improve benefit, the other alternative pathways lead to resistance. Moreover, the balance between therapeutic efficacy and toxicity must be evaluated before administration to patients. Another therapeutic strategy consists in targeting BMDCs or pericytes and CAFs in addition to tumor cells. This approach seems relevant since BMDCs and local stromal cells blockade leads to an impairment of tumor growth (Bergers and Hanahan, 2008; Crawford and Ferrara, 2009; Liu et al., 2010). The treatment of patients with diffuse-type giant tumor cells with a CSF-1 antibody elicits objective response (Ries et al., 2014). This result raises the possibility to combine this antibody to anti-angiogenic agents.

Another promising therapeutic strategy consists in targeting lymphangiogenesis and angiogenesis. Lymphangiogenesis induced by anti-angiogenesis dedicated compounds gives rise to node metastasis, leading at term to an increased metastatic rate and poor outcome in patients (Dufies et al., 2017a). Moreover, these lymphatic vessels play a key role in the cancer-induced immune tolerance. Indeed, tumor associated lymphatic vessels upregulated Program-Death Ligand 1 (PDL1) inhibiting T cell activation and therefore anti-tumor response (Dieterich et al., 2017). Recently, the anti-PDL1 antibody, avelumab combined with axitinib was compared to sunitinib for advanced RCC. The progression free survival was 13.8 months and significantly higher than sunitinib alone (8.4 months) (Motzer et al., 2019). A phase III study comparing the anti-PDL1 antibody, atezolizumab, plus bevacizumab versus sunitinib was assessed in metastatic RCC and confirmed these results (Rini et al., 2019b, 3). Among the tested combinations, the anti-PD1, pembrolizumab plus axitinib combo improved the PFS but also the OS of RCC patients (Rini et al., 2019a).

Nevertheless, despite their effects on PFS and OS, these combinations are not curative. The development of animal models mimicking the tumor microenvironment as well as preclinical evaluations of combo therapies are urgently needed to improve patients PFS and OS. To reach the “Golden Age” of tumor treatment as defined by Hsieh et al. (2017) new treatment options are needed either to improve the therapeutic effects of anti-angiogenics and immunotherapies or by inhibiting new relevant pathways involved in innate refractoriness or acquired resistance. The current anti-cancer strategies are based on the inhibition of a specific target playing a key role in tumor development [example: EGFR (lung cancers); HER2 (breast cancers); BRAF (melanoma)]. Because of relapses on these strategies, combinations with conventional chemotherapy [taxanes (breast) platin salts (lung)] or other targeted therapies like anti-angiogenics or immunotherapies have entered in the therapeutic arsenal. However, the second strategy often combined different toxicities and cannot be administered at long terms, limiting the therapeutic index. However, the “magic bullet” does not exist because cancers integrate several mechanisms of evasion to one treatment. Hence, instead of inhibiting several targets with several drugs, the ideal strategy relies on the use of one inhibitor targeting multiple hallmarks of cancers, i.e., tumor cell proliferation/stemness, angiogenesis, chronic inflammation, and immune tolerance.

By destroying the vascular network, antiangiogenic therapies efficacy should have cause vascular network destruction leading to tumor cells asphyxia and nutrient starvation. Moreover, antiangiogenic treatments should have targeted only normal endothelial cells that cannot undergo genetic plasticity, a specific property of tumor cell adaptation to treatments. However, aberrant expression, by tumor cells, of receptors inhibited by antiangiogenic drugs stimulated the genetic adaptation of tumor cells mainly through epigenetic modifications. For example, EZH2 a specific histone methyl transferase is a driver of sunitinib resistance in kidney cancers (Adelaiye-Ogala et al., 2017). In addition to tumor cells, tumor endothelial cells undergo epigenetic modifications crucial for adaptation to the antiangiogenic therapies (Ciesielski et al., 2020).

The correlation between the efficacy of antiangiogenic drugs and tumor grade was also a neglected parameter. Controversial results emerged from their efficacy in non-metastatic versus metastatic kidney cancers. Whereas they are the standard of cancer for metastatic tumors their efficacy as an adjuvant therapy gave conflicting results. The ASSURE trial (NCT00326898) showed no survival benefit relative to placebo whereas the S-TRAC trial showed that sunitinib in an adjuvant setting prolonged the disease free survival for more than 1 year (Haas et al., 2016; Ravaud et al., 2016). These complex features supposed that anti-angiogenic drugs affect other cells than ECs. Hence, the drugs indirectly affect immune cells. Sunitinib for example reverses immune suppression (Finke et al., 2008). In this process, myeloid derived suppressor cells are one of the main targets of sunitinib (Ko et al., 2009). Moreover, inhibition of VEGFA or VEGFR decreased the expression of immune checkpoints involved in immune tolerance, by T cells (Voron et al., 2015). Hence, the crosstalk between angiogenesis and immune cells explain the efficacy of combining antiangiogenic drug to immune checkpoint inhibitors (Motzer et al., 2019; Rini et al., 2019b). Immunetolerance is most of the time encountered in advanced tumors in which angiogenesis is key for metastatic spreading. The relevance of inhibiting angiogenesis was based on these extreme cases. However, blood or lymphatic vessels vehiculate active cytotoxic immune cells to prevent the development of low-grade tumors that did not undergo immune tolerance. Hence, favoring the development of lymphatic vessels through injection of VEGFC decreased the growth of experimental glioblastoma by enabling immunosurveillance (Song et al., 2020). Hence, these experiments completely revisited the notion that angiogenesis is systematically detrimental. The hypothesis that vessels must be normalized in cancer had emerged during the last decade (Goel et al., 2011). This hypothesis stipulates that normalization of tumor vessels will shape the tumor microenvironment leading to the control of tumor progression and to the improvement of the therapeutic response (Martin et al., 2019).

With the advent of the immunotherapy, the blockage of angiogenesis should be reconsidered and the “blasting missil” must be discovered.

It is now evident that targeting only one mechanism involved in cancer development is insufficient. The cancer Hallmarks described by Hanahan and Weimberg probably shape the future treatments to increase the percentage of complete remissions. What is the ideal strategy? Targeting at the same time different Hallmarks with already approved therapies or to find targets that drive concomitantly the different Hallmarks? If these targets exist, a specific inhibitor will serve as a “blasting missile” to destroy the tumor. The reality is probably an intermediate option.

## Author Contributions

CM and GP are equally responsible for all parts of the manuscript. All authors contributed to the article and approved the submitted version.

## Conflict of Interest

The authors declare that the research was conducted in the absence of any commercial or financial relationships that could be construed as a potential conflict of interest.
